# Effects of prophylactic antibiotics before peritoneal dialysis catheter implantation on the clinical outcomes of peritoneal dialysis patients

**DOI:** 10.1080/0886022X.2019.1568259

**Published:** 2019-02-01

**Authors:** Xihui Liu, Xiaoyan Zuo, Xia Sun, Zhao Hu

**Affiliations:** a Department of Nephrology, Shandong University Qilu Hospital, Jinan, China;; b Department of Nephrology, Linyi People’s Hospital, Linyi, China;; c Department of Nephrology, Yiyuan People’s Hospital, Zibo, China

**Keywords:** Peritoneal dialysis, peritoneal dialysis catheter, prophylaxis, antibiotics, peritonitis

## Abstract

**Background:** Peritoneal dialysis (PD) related infections, such as peritonitis, are still the main obstacle for the development of PD. Prophylactic antibiotic as one of the interventions to prevent early peritonitis was recommended to use before PD catheter insertion by International Society for Peritoneal Dialysis (ISPD) guidelines, In our hospital, however, since 2012, the prophylactic antibiotics for insertion of PD catheters were not allowed to use because of our hospital’s regulation. In order to analyze the outcomes of PD patients without using prophylactic antibiotics before the PD catheter insertion, we compared the PD patients with or without prophylactic antibiotics before PD catheter insertion.

**Methods:** This retrospective study included 247 patients undergoing permanent PD catheter placement with conventional open surgical method consecutively between February 2008 and June 2013. Of these, 154 patients were given intravenous cefazolin, 1.0 g, 0.5–2 h before the procedure (antibiotic group) and 93 patients were not given prophylactic antibiotics (nonantibiotic group). All the patients were administered intermittent PD within 24 h after PD catheter insertion. The early complications and long-term outcomes were recorded respectively.

**Results:** There was no significant difference in the incidence of peritonitis and exit-site/tunnel infection and mechanical complications between the two groups in the first 30 days after the PD catheter implantation. In addition, after 6 years of follow-up, no difference was seen between the two groups in patient survival, technique survival, and peritonitis-free survival.

**Conclusions:** Our study does not show any beneficial effect of antibiotic prophylaxis in reducing the postoperative peritonitis.

## Introduction

Peritoneal dialysis (PD) is one of the important renal replacement therapies for patients with end-stage renal disease (ESRD) and is used in more than 200 000 such patients globally [1]. It is known that PD has more clinical advantages compared with hemodialysis (HD) such as improved survival the first years after initiation of dialysis, the better quality of life, and the better protection of residual renal function [2]. In addition, PD allows a patient to live a life more fully, and it is a cost-effective modality [3]. PD should be used more frequently than it is, but the perceived risk of peritonitis is a major impediment to its wider application [4–6], peritonitis also leads to technique failure, increased hospitalization, and increased mortality [7–9]. Correspondingly, a number of interventions to prevent peritonitis based primarily on antimicrobial prophylaxis and modification of the PD catheter and system in PD patients have been put into effect. With respect to antimicrobial strategies, perioperative intravenous antibiotics compared with no treatment significantly reduced the risk of early peritonitis. Similarly, several catheter-related strategies such as surgical approaches for the insertion of the PD catheter (laparoscopy vs. laparotomy), types of PD catheters (straight vs. coiled), and types of PD sets (Y-set vs. twin-bag system) have been developed to evaluated the efficacy, benefits, and harms for the prevention of peritonitis in PD patients [4]. In our hospital, the PD catheters were implanted using open surgical method and the prophylactic antibiotics were also administrated before the PD catheter insertion according to the International Society for Peritoneal Dialysis (ISPD) and CARI guidelines recommendation [10,11] before 2012. However, since 2012, the prophylactic antibiotics for insertion of PD catheters were not allowed to use because of our hospital’s regulation, so the retrospective study was performed in order to evaluate the clinical outcomes of our patients with newly placed PD catheters without using preoperative antibiotic prophylaxis at our center.

## Materials and methods

### Patients

This was a single-center retrospective study conducted at Linyi People’s Hospital in Linyi City, China. 247 patients with ESRD undergoing permanent PD catheter placement consecutively from February 2008 to June 2013 were enrolled in this study. The exclusion criteria were patients younger than 18 years. The patients were divided according to whether or not to use antibiotic prophylaxis into antibiotic group and nonantibiotic group. Demographics and comorbidities were recorded including age, gender, weight, presence of diabetes and cardiovascular disease, and the primary disease of ESRD. Biochemical variables before the procedure including hemoglobin (Hb), serum albumin (Alb), serum potassium (K), serum sodium (Na), serum bicarbonate (HCO_3_), serum calcium (Ca), serum phosphorus (P), blood urea nitrogen (BUN), and serum creatinine (Scr) were also collected.

### Preoperative preparation

All the patients were hospitalized to the nephrology ward from the clinic of nephrology. If they are eligible for PD and have no contraindications for PD catheter insertion, the written informed consent is obtained from the patients or relatives. All the PD catheters are placed by one same designated experienced nephrologist using the same conventional open surgical technique during all the study period.

### The open surgical procedure

Straight, double-cuffed Tenckhoff 41 cm silicon catheters were used in all the patients, and all the surgical procedure were implemented under local anesthesia in the central operative unit. The process is performed according to the description in the second edition textbook of PD [12], however, the insertion site of our patients was located at paramedian incision distance from the upper border of the pubic symphysis 11–12 cm depending on the size of the patients, the position should be 12–13 cm (above the umbilicus) in small abdomen patients. Apart from the purse-string suture tied securely to fix the catheter in the posterior rectus sheath and peritoneum, another method to fix the catheter which is made between two sutures in the anterior rectus sheath where the catheter is passed through has also been made, after the two sutures are securely tied respectively it is just like a purse-string to fix the catheter tightened. And the inner cuff is buried among the rectus muscle fibers.

### Postoperative care

After PD catheter implantation, intermittent low dose PD in the supine position was initiated within 24 h without break-in period for all the patients, and the dialysis prescription was administered a fill volume of 1000 mL, a dwell time of 3 h, and frequency of 4 times daily. The twin-bag connection system was used for all patients. The dressing changes were done routinely by the nephrologist in the third day after the operation so that the incision infection and incision fat liquefaction were judged and early addressed. The dressing changes of incision and exit site were done twice a week before the incision was healed. Suture removal was performed 7–10 days after catheter implantation. The training program on the PD technique for the patient was also started in the third day after insertion of the catheter by a trained PD nurse. After 10–14 days of treatment, the uremia symptoms were eliminated and the patients were discharged, and the patients were converted to continuous ambulatory peritoneal dialysis with full-volume 2000 mL dialysate exchange.

### Clinical outcomes

The primary outcome measure was early complications which were examined in the first 30 days following the insertion of PD catheter. Infectious complications included peritonitis, exit-site/tunnel infection. Mechanical complications included leakage, catheter blockage, and catheter migration. Complications were defined in accordance with the ISPD guidelines [11,13]. Peritonitis was defined as the presence of at least two of the following conditions: abdominal pain or tenderness; presence of white blood cells (≥100 cells/mL) in the peritoneal effluent, with at least 50% polymorphs; and positive dialysate culture results. The secondary outcome measures were long-term outcomes including technique survival, patient survival, and peritonitis-free survival (time to first episode of peritonitis) which were recorded following the catheter insertion until the censored time or the end of the study. In the technique survival analysis, permanent conversion to HD was a final event and patients were censored at other events. In the patient survival analysis, only death was considered as a final event and patients were censored when PD was stopped for any other reason, including transfer to HD, renal transplantation, recovery of renal function, or at the end of the observation period. We analyzed the outcome following catheter placement until February 2014 or until discontinuation of PD.

### Statistical analysis

Values for continuous variables are presented as mean ± standard deviation. Categorical variables are expressed as a percentage or frequencies. Values of categorical variables were compared using the chi-square test or Fisher’s exact test; the Student’s *t*-test was used for continuous variables. The Kaplan–Meier method and log-rank test were used to examine the technique survival, patient survival, and peritonitis-free survival by comparing antibiotic group with nonantibiotic group. All *p* values are two-tailed and statistical significance was assumed at a *p* value of less than 0.05. Data were analyzed using the SPSS software package (version 17.0; SPSS, Chicago, IL).

## Results

### Baseline characteristics

Two hundred and forty-seven ESRD patients (154 antibiotic, 93 nonantibiotic) were included. The baseline characteristics are shown in Table 1. Of the 247 patients, 120 were men (48.6%), 127 were women (51.4%), with a mean age of 52.6 ± 14.9 years. The primary diseases were chronic glomerulonephritis, diabetic nephropathy, hypertension, obstructive nephropathy, polycystic kidney disease, and others including interstitial nephritis, secondary nephritis, renal cancer, and unknown. There was no significant difference in gender, age, weight, primary disease, comorbidities presented as diabetes mellitus, cardiovascular disease, cerebrovascular disease, and hypertension, as well as laboratory values between the two groups.

**Table 1.** Baseline characteristics.

Characteristic	Antibiotic	Nonantibiotic	*p* value
Number of patients (%)	154 (62.3)	93 (37.7)	
Age (years)	52.5 years	52.7 years	0.911
Gender [male (%)]	77 (50.0)	43 (46.2)	0.566
Primary disease, n (%)			
CGN	93 (60.4)	55 (59.1)	0.846
Diabetic nephropathy	42 (27.3)	26 (28.0)	0.907
Hypertension	3 (1.9)	2 (2.2)	1.000
Obstructive nephropathy	4 (2.6)	3 (3.2)	1.000
PCKD	6 (3.9)	2 (2.2)	0.714
Others	6 (3.9)	5 (5.4)	0.820
Comorbid disease, n (%)			
Cardiovascular disease	23 (14.9)	10 (10.8)	0.349
Cerebrovascular disease	18 (11.7)	7 (7.5)	0.293
Diabetes mellitus	43 (27.9)	27 (29.0)	0.851
Hypertension	47 (30.5)	22 (23.7)	0.244
Weight (kg)	62.4 ± 11.2	61.1 ± 11.1	0.411
Laboratory values			
Hb (g/L)	80.6 ± 20.1	80.4 ± 18.3	0.943
Alb (g/L)	31.3 ± 5.7	31.7 ± 5.8	0.564
K (mmoL/L)	4.78 ± 1.17	4.82 ± 1.04	0.797
Na (mmoL/L)	136.86 ± 5.21	137.29 ± 3.94	0.498
HCO_3_ (mmoL/L)	16.96 ± 4.73	16.81 ± 4.51	0.813
Ca (mmoL/L)	1.92 ± 0.28	1.90 ± 0.29	0.530
P (mmoL/L)	2.36 ± 0.76	2.37 ± 0.81	0.934
BUN (mmoL/L)	38.9 ± 14.7	39.4 ± 16.7	0.807
Scr (umoL/L)	883.9 ± 352.1	930.2 ± 420.7	0.359

PCKD: policystic kidney disease; CGN: chronic glomerulonephritis; Hb: hemoglobin; Alb: serum albumin; K: serum potassium; Na: serum sodium; HCO_3_: serum bicarbonate; Ca: serum calcium; P: serum phosphorus; BUN: blood urea nitrogen; Scr: serum creatinine.

### Early complications after implantation

As shown in Table 2, seven patients, three in antibiotic group and four in nonantibiotic group, developed fat liquefaction of postsurgical incision, and one of the three patients with fat liquefaction in antibiotic group presented wound infection and *Staphylococcus albus* was cultured from exudate of the wound. These fat liquefactions of incisions were all healed after 14–16 days by exudate drainage and dressing change every day. There was no difference in the rate of incision fat liquefaction between the two groups (*p* = 0.431). In this study, three patients in antibiotic group and one patient in nonantibiotic group presented exit-site infection (ESI) within 1 month, difference between the two groups was not found either (*p* = 1.000). Five patients in antibiotic group (3.2%) and six patients in nonantibiotic group (6.5%) developed peritonitis within the first 30 days after catheter insertion (*p* = 0.387). One of the five patients in antibiotic group developed peritonitis in the first dialysate exchange and suffered from microbial growth with *Staphylococcus epidermidis*, and catheter removal was performed due to refractory peritonitis and concurrent outflow failure and switched to permanent HD. However, one of the six patients in nonantibiotic group developed refractory peritonitis cultured with *Pseudomonas aeruginosa* in the 22nd day after catheter insertion and re-hospitalized for catheter removal and transferred to permanent HD. No early tunnel infection occurred in the two groups. As for early mechanical complications, one patient in antibiotic group developed omental wrapping with inflow and outflow dysfunction in the second day after catheter implantation, at the same time, catheter tip migration out of the true pelvis was also found in this patient by abdominal radiographies, after bridging HD, the catheter repair and small part of omentectomy was carried out for the patient and the catheter worked well throughout the study period. Another patient developed catheter blockage in nonantibiotic group in the sixth day after catheter insertion, which was resolved after sealing the PD catheter with urokinase. However, there was no early pericatheter leakage in the study patients.

**Table 2.** Complications within the first 30 days of catheter insertion.

	Antibiotic	Nonantibiotic	*p* value	Total
Number of patients (%)	154 (62.3)	93 (37.7)		
Surgical incision (%)				
Incision fat liquefaction	3 (1.9)	4 (4.3)	0.431	7 (2.8)
Exit-site infection (%)	3 (1.9)	1 (1.1)	1.000	4 (1.6)
Peritonitis (%)				
within 1 month	5 (3.2)	6 (6.5)	0.387	11 (4.5)
during hospitalization period	2 (1.3)	2 (2.2)	0.633	4 (1.6)
from being discharged to 1 month	3 (1.9)	4 (4.3)	0.431	7 (2.8)
Early catheter loss (%)	1 (0.6)	1 (1.1)	1.000	2 (0.8)
Mechanical complications (%)				
Omental wrapping and migration	1 (0.6)	0	1.000	
Catheter blockage	0	1 (1.1)	0.372	
Drain problem	0	1 (1.1)	0.372	
Dialysate leakage	0	0		

### Follow-up outcomes of the study patients

**Figure 1.**
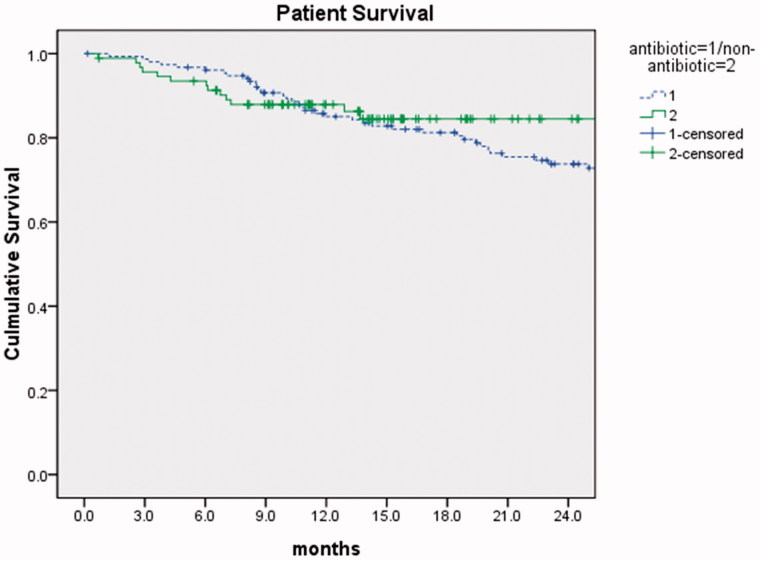
Culmulative survival for antibiotic patients compared with nonantibiotic patients. *p* = 0.505, the log rank test.

The mean follow-up period was 21.7 ± 13.7 months (range: 1.3–72.6 months). At the end of the study period, 153 patients (61.9%) were still on PD, 94 patients (38.5%) dropped out due to various causes, including 22 patients transferred to HD, 9 patients received transplantation, 58 patients died, and 5 patients lost their visit (Table 3). Of the 58 patients who died, causes of death included cardiovascular disease (15 patients), cerebrovascular disease (8 patients), cancer (4 patients), psychosocial factors (5 patients), and others (26 patients) including withdrawing treatment, and multiple organ failure or unknown reasons. Among these patients, 16 died due to peritonitis directly or indirectly. Kaplan–Meier analysis of patient survival at 1 year and 2 years postcatheter insertion showed no difference between the two groups. The probability of patient survival in antibiotic and nonantibiotic group was 85% and 87.9% at 1 year, 73.8% and 84.5% at 2 years, respectively (*p* = 0.505, Figure 1).

**Table 3.** Long-term follow-up clinical outcomes.

	Antibiotic	Nonantibiotic	*p* value	Total
Number of patients (%)	154 (62.3)	93 (37.7)		
Follow-up (months)	26.1 ± 14.9	14.2 ± 6.1	0.000	21.7 ± 13.7
Exit-site/tunnel infection (%)	3 (1.9)	0	0.296	
Number of peritonitis episodes (%)	64 (41.6)	32 (34.4)	0.264	96 (38.9)
1 episode, n (%)	37 (24.0)	20 (21.5)	0.649	57 (23.1)
2 episodes, n (%)	6 (3.9)	5 (5.4)	0.820	11 (4.5)
3 or more than 3 episodes, n (%)	21 (13.6)	7 (7.5)	0.142	28 (11.3)
Outcome				
Still on PD, n (%)	80 (51.9)	73 (78.5)	0.000	153 (61.9)
Death, n (%)	45 (29.2)	13 (14.0)	0.006	58 (23.5)
Transplantation, n (%)	7 (4.5)	2 (2.2)	0.533	9 (3.6)
Transfer to HD, n (%)	17 (11.0)	5 (5.5)	0.130	22 (8.9)
Lost visit, n (%)	4 (2.6)	1 (1.1)	0.653	5 (2.0)

Twenty-two patients transferred to HD over the study period, 17 (11.0%) in antibiotic group, and 5 (5.5%) in nonantibiotic group (*p* = 0.130). The rate of technique survival for antibiotic and nonantibiotic patients were 95.7% and 93.9% at 1 year, 91.9% and 93.9% at 2 years, respectively (*p* = 0.848). Among the 22 patients switched to HD, 13 were directly caused by peritonitis, and 3 of the 13 patients were fungal peritonitis.

### Late infectious complications

There were three patients developed severe exit-site/tunnel infection need to hospitalize for treatment in antibiotic group. However, there were no such patients in nonantibiotic group. Of the three patients, one occurred in the 8.3 month after the catheter insertion and cultured positive organism with *Pseudomonas aeruginosa*, one occurred in the 9.9 month after the catheter insertion, but no purulent exudate sample was sent to culture, another occurred in the 40.5 month after the catheter insertion and cultured positive organism with *Staphylococcus aureus*. They were all cured and then discharged.

Table 3 shows that there were 96 of all the study patients (38.9%) with one or more episodes of peritonitis, and no significant difference in regard to the number of peritonitis episodes was found between the two groups (*p* = 0.264). No exit-site/tunnel infection related peritonitis or peritonitis related exit-site/tunnel infections occurred during all study period.

**Figure 2.**
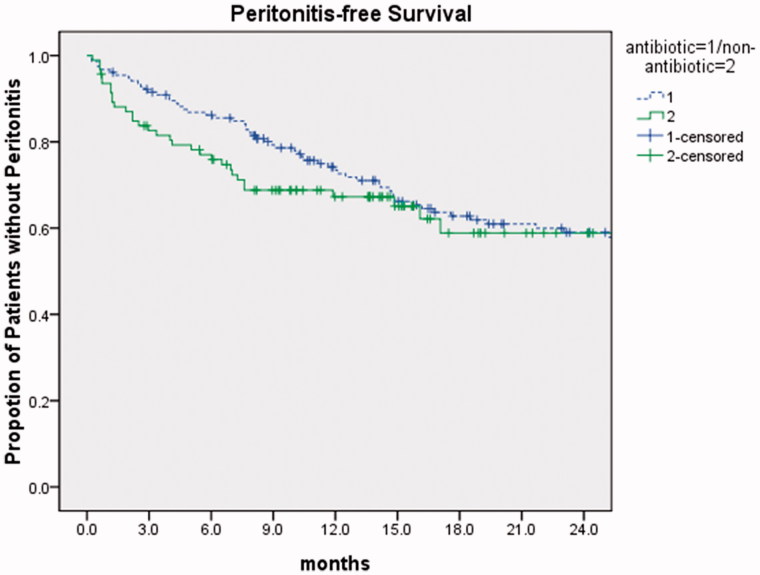
Culmulative peritonitis-free survival for antibiotic patients and for nonantibiotic patients. *p* = 0.246, the log rank test.

No significant difference was found between antibiotic group and nonantibiotic group regarding peritonitis-free survival evaluated by Kaplan–Meier method. The proportion of antibiotic and nonantibiotic patients free from peritonitis were 74.2% and 67.3% at 1 year, 59.0% and 58.9% at 2 years, respectively (*p* = 0.426, Figure 2).

## Discussion

The present study has demonstrated there were only a few early complications via early initiation of dialysis after surgical implantation of PD catheters with or without using preoperative prophylaxis antibiotics in our hospital. Antibiotic prophylaxis refers to the administration of short-term antibiotics in surgical patients before the initiation of surgery, and the decision to administer antibiotics should be made by considering their risk and benefits. One of them includes utilization of the NNIS (National Nosocomial Infections Surveillance) score system, which considers three factors, including classification of the operation based on wound surgery, the patient’s co-morbid medical condition, reflected by physical status of the surgical patients according to ASA (American Association of Anesthesiologist), and duration of the operation according to the NNIS survey [14]. As we know, antibiotic prophylaxis is not recommended for clean surgical wound, except for a foreign body is left within the wound, which will increase the risk of infection [14,15]. Since the operation for insertion of a PD catheter can be classified as clean surgery with implantation of foreign body [16]. The administration of prophylactic antibiotics is reasonable. The ISPD guidelines recommended prophylactic antibiotics should be used before PD catheter implantation [11,13]. Some studies also confirmed the role of prophylactic antibiotics before PD catheter insertion in preventing postoperative infection. In prospective studies, Wikdahl et al. [15] randomized 38 patients, Bennett-Jones et al. [18] randomized 26 patients, Gadallah et al. [17] randomized 221 patients, and showed a significant reduction in the incidence of peritonitis, with short periods of less than 4 weeks [15,17,18]. One of the studies showed an advantage of prophylactic vancomycin compared with cefizolin [17]. Although vancomycin prophylaxis has been demonstrated to be effective, its routine use is not recommended [11]. In retrospective studies, Golper et al. [19] in the Network 9 Study, showed a 39% reduction in the risk of peritonitis and a 38% reduction in combined peritonitis and exit-site/tunnel infection. Sardegna et al. [20] showed significant benefit using multiple antibiotics in a pediatric dialysis population. The patients in our unit were routinely given prophylactic antibiotics before PD catheter implantation. Since 2012, however, prophylactic antibiotics prior to the operation were stopped because of hospital’s regulation. It is interesting that there is no benefit of early infection in the antibiotic patients compared with nonantibiotic patients in our study, which consents with the results of several studies. For instance, Lye et al. [21] randomized 50 patients and showed no benefit from antibiotic prophylaxis with single-dose cefazolin and gentamicin, compared with no antibiotics, on the incidence of peritonitis up to 3 months postcatheter insertion. The United State Renal Data Systerm (USRDS) [22] 1992 Data Report showed in 3366 patients on home PD in 1989 that there was no difference in peritonitis between patients who had received antibiotic prophylaxis or not. There are some possible explains for our result. Firstly, although the silicon catheter represents a foreign body or foreign material, it is not just like the two conditions for the clean surgical wound in the paper which was reported by Budi Setiawan [14] that included the insertion of intravascular prosthetic material or for any operation in which surgical site infection would pose catastrophic risk such as all cardiac operation, arterial graft placement at extremities, and neurosurgical operation. For the PD patients, the catheter is inserted to the abdominal pelvis rather than to the intravascular, and the outcome of surgical site infection cannot be lethal. Secondly, the lateral incision site in our patients is only 2–3 cm long and there are two purse-strings in the posterior rectus sheath and anterior rectus sheath respectively to anchor the inner cuff among the rectus muscle, which plus outer cuff just like three barriers to prevent the pericatheter leakage and the tunnel infection or tunnel infection related peritonitis. In the meanwhile, exit-site care was done with iodide by the experienced nephrologist in the ward and a mupirocin ointment was given each patient before being discharged to prevent or treat the ESI or suspected infection if redness and purulent exudate of exit-site area was found at home. Thirdly, the duration of operation for PD catheter insertion is all within 1 h. In addition, the PD catheter was extracted out through an exit site but not directly through the incision site. Therefore, it seems that the PD catheter has no effect on wound healing.

From the study, seven patients (2.8%) were found to have postoperative complications with fat liquefaction of the surgical incision in the study cohort. Of these patients, five were found with incision fat liquefaction in the third day and two in the eighth day, so the dress change of the incision in the third day routinely is essential and important. It is known that fat liquefaction is the necrosis of adipose tissue without infection which is the main cause of prolonged healing of aseptic postsurgical incision [23], in our patients without fat liquefaction, the incision healing and suture removal were 7–10 days, however, in the patients with fat liquefaction, the incision was addressed 14–16 days after the incision fat liquefaction was found till wound healing, and it prolongs the stay in the hospital. It is reported that factors contributing to fat liquefaction in postoperative wounds are more often in overweight or obesity patient with thick abdominal subcutaneous fat or overuse of electrotome in surgery [23]. Similarly, our seven patients who occurred fat liquefaction are all female and with thick subcutaneous fat. We have gotten more experience to prevent the occurrence of fat liquefaction of incision in the future operation from the seven patients. First, the subcutaneous tissue and fat layers were bluntly dissected down to the anterior rectus sheath instead of using electrotome. Second, when the thick subcutaneous fat layer is sutured, the fat layer will be stitched one after another completely, after which the suture is tied one by one to avoid leaving a dead cavity in the fat layer.

In our center, all the patients started PD without break-in period due to the poor economic status and therefore late start of dialysis. Many studies have reported leakage occurs more frequently during the immediate postoperative period in early initiation or immediate start PD [24,25], because dialysis immediately postcatheter insertion could impair normal tissue healing, causing or contributing to an increased dialysate leakage or tunnel infection rate [17]. However, no leakage was found within 30 days or during the study period in our study. Yang et al. also reported a low leakage rate (2.2%) within 6 months in early start PD patients [26]. The reasons for the zero incidence rate of pericatheter leakage in our PD patients, we think, should be owed to some following aspects. One is the experienced and skillful operation technique by the same nephrologist; another is the two purse-string sutures and the two cuffs as the barriers mentioned above already to prevent the leakage occur; in addition, low dialysate volume and with a supine position during the dialysis commencement within 14 days may lower the intraperitoneal pressure. These abovementioned strategies were also described by others [27–29]. Therefore, immediate start dialysis within 24 h after PD catheter insertion with surgical method may be feasible, which can decrease the need of bridging HD with a temporary central venous catheter which could increase the overall complications related to the HD catheter use. Similarly, in our study patients, the bridging HD was definitely avoided after early initiation of dialysis.

The overall all caused mortality rate is 23.5% (58 patients), 45 (29.2%) in antibiotic group, and 13 (14.0%) in nonantibiotic group (*p* = 0.006). The low mortality rate of nonantibiotic patients derived from the duration of short follow-up period with only 2 years, instead, 6 years in the antibiotic patients.

Although Gadallah et al. [17] considered that peritonitis occurred after 14 days from the date of the procedure was not related to the primary procedure. Lye et al. [21] felt that antibiotic prophylaxis would not be expected to prevent infections after 4 weeks of the surgical procedure. There are still 29 patients accounting for 30.9% of all dropout patients have dropped out due to peritonitis directly or indirectly. So every effort should be made to prevent the occurrence of peritonitis.

We believe that as the number of new patients with PD catheter insertion increased, subsequently the experience of our nephrology and nurse team increased too, and it is well known the catheter tend to survive better over time [30]. The PD catheter insertions in the nonantibiotic patients of our center were preformed since 2012. Although without antibiotic prophylactic, the early infectious complication and the follow-up outcomes are no worse than that in antibiotic group.

The present study has several characteristics. First, the study is related to the hospital regulation which cannot be found in other studies. Second, it is a single-center and retrospective study, which limited conclusions drawn from this study. But we still analyzed the long-term outcomes which may not be related to the prophylactic antibiotics before the operation. Finally, all dialysis are initiated within 24 h after the catheter placement, which may contribute to the early infectious complications, however, there are only four patients (1.6%) developed peritonitis during the hospitalization period, other seven patients (2.8%) occurred peritonitis from being discharged to 1 month after the catheter insertion (Table 2). Three of the four patients during the hospitalization period occurred peritonitis after seven postoperative days, suggesting that in the three patients, introduction of bacterial into the peritoneum was not from surgical contamination in which case peritonitis would occur fairly soon after catheter insertion but from another source [20], most likely from contamination at one of the connection points during the dialysate exchange in our patients.

Overall, although there have been four randomized prospective studies addressing the issue of whether prophylactic antibiotics before the insertion of PD catheter reduce peritonitis, only one of the studies is larger samples. Further, well designed large prospective randomized controlled trials should be needed to get more evidence with regard to the benefit of antibiotic prophylaxis to the postoperative infections.

In summary, we conclude that we cannot see any beneficial effect of antibiotic prophylaxis in reducing the postoperative peritonitis for patients undergoing insertion of PD catheter.

## References

[1] Campbell D, Mudge DW, Craig JC, et al. Antimicrobial agents for preventing peritonitis in peritoneal dialysis patients. Cochrane Database Syst Rev. 2017;4 pages. CD004679. 28390069 10.1002/14651858.CD004679.pub3 PMC6478113

[2] Tokgoz B Clinical advantages of peritoneal dialysis. Perit Dial Int. 2009;29:S59–S61. 19270233

[3] Morton RL, Tong A, Webster AC, et al. Characteristics of dialysis important to patients and family caregivers: a mixed methods approach. Nephrol Dial Transplant.2011;26:4038–4046. 21482637 10.1093/ndt/gfr177

[4] Bonifati C, Pansini F, Torres DD, et al. Antimicrobial agents and catheter-related interventions to prevent peritonitis in peritoneal dialysis: using evidence in the context of clinical practice. Int J Artif Organs. 2006;29:41–49. 16485238 10.1177/039139880602900103

[5] Diaz-Buxo JA Modality selection. J Am Soc Nephrol. 1998;9:S112–S117. 11443757

[6] Piraino B, Bernardini J, Sorkin M Catheter infections as a factor in the transfer of continuous ambulatory peritoneal dialysis patients to hemodialysis. Am J Kidney Dis. 1989;13:365–369. 2719024 10.1016/s0272-6386(89)80018-6

[7] Churchill DN, Thorpe KE, Vonesh EF, et al. Lower probability of patient survival with continuous peritoneal dialysis in the United States compared with Canada. Canada-USA (CANUSA) Peritoneal Study Group. J Am Soc Nephrol. 1997;8:965–971. 9189865 10.1681/ASN.V86965

[8] Digenis GE, Abraham G, Savin E Pertonitis-related deaths in continuous ambulatory peritoneal dialysis (CAPD) patients. Perit Dial Int. 1990;10:45–47. 2085582

[9] Annigeri R, Conly J, Vas S Emergence of mupirocin resistant Staphylococcus aureus in chronic peritoneal dialysis patients using mupirocin prophylaxis to prevent exit-site infection. Perit Dial Int. 2001;21:554–559. 11783763

[10] Bannister K, Walker A, Lonergan M, et al. Evidence for peritonitis treatment and prophylaxis. CARI guidelines. Nephrology (Carlton). 2004;9:S72–S75. 15469562 10.1111/j.1440-1797.2004.00303.x

[11] Keane WF, Baile GR, Boeschoten E, et al. International Society for Peritoneal Dialysis, adult peritoneal dialysis related peritonitis treatment recommendations: 2000 Update. Perit Dial Int. 2000;20:396–411. 11007371

[12] Twardowski ZJ, Nichols WK Peritoneal dialysis access and exit-site care including surgical aspects In: Gokal R, Khanna R, Krediet RT, Nolph KD, editors. Textbook of peritoneal dialysis. Dordrecht: Springer; 2000 p. 333–338.

[13] Li PK, Szeto CC, Piraino B, et al. Peritoneal dialysis-related infections recommendations: 2010 update. Perit Dial Int. 2010;30:393–424. 20628102 10.3747/pdi.2010.00049

[14] Setiawan B The role of prophylactic antibiotics in preventing perioperative infection. Acta Med Indones. 2011;43:262–266. 22156360

[15] Wikdahl AM, Engman U, Stegmayr BG, et al. One-dose cefuroxime i.v. and i.p. reduces microbial growth in PD patients after catheter insertion. Nephrol Dial Transplant. 1997;12:157–160. 9027792 10.1093/ndt/12.1.157

[16] Katyal A, Mahale A, Khanna R Antibiotic prophylaxis before peritoneal dialysis catheter insertion. Adv Perit Dial. 2002;18:112–115. 12402600

[17] Gadallah MF, Ramdeen G, Mignone J, et al. Role of preoperative antibiotic prophylaxis in preventing postoperative peritonitis in newly placed peritoneal dialysis catheters. Am J Kidney Dis. 2000;36:1014–1019. 11054359 10.1053/ajkd.2000.19104

[18] Bennett-Jones DN, Martin J, Barratt AJ, et al. Prophylactic gentamicin in the prevention of early exit-site infections and peritonitis in CAPD. Adv Perit Dial. 1988;4:147–150.

[19] Golper TA, Brier ME, Bunke M, et al. Risk factors for peritonitis in long-term peritoneal dialysis: the Network 9 peritonitis and catheter survival studies. Academic Subcommittee of the Steering Committee of the Network 9 Peritonitis and Catheter Survival Studies. Am J Kidney Dis. 1996;28:428–436. 8804243 10.1016/s0272-6386(96)90502-8

[20] Sardegna KM, Beck AM, Strife CF Evaluation of perioperative antibiotics at the time of dialysis catheter placement. Pediatr Nephrol. 1998;12:149–152. 9543378 10.1007/s004670050427

[21] Lye WC, Lee EJ, Tan CC Prophylactic antibiotics in the insertion of Tenckhoff catheters. Scand J Urol Nephrol. 1992;26:177–180. 1626207 10.1080/00365599.1992.11690450

[22] United States Department of Health and Human Services Public Health Service, National Institutes of Health, National Institute of Diabetes and Digestive and Kidney Disease, USRDS Annual Data Report. Catheter-related factors and peritonitis risk in CAPD patients. Bethesda (MD): United States Renal Data System; 1992.

[23] Shi Z, Ma L, Wang H, et al Insulin and hypertonic glucose in the management of aseptic fat liquefaction of post-surgical incision: a meta-analysis and systematic review. Int Wound J. 2013;10:91–97. 22325039 10.1111/j.1742-481X.2012.00949.x PMC7950496

[24] Diaz-Buxo JA Mechanical complications of chronic peritoneal dialysis catheters. Semin Dial. 2007;4:106–111.

[25] Banli O, Altun H, Oztemel A Early start of CAPD with the Seldinger technique. Perit Dial Int. 2005;25:556–559. 16411521

[26] Yang YF, Wang HJ, Yeh CC, et al. Early initiation of continuous ambulatory peritoneal dialysis in patients undergoing surgical implantation of Tenckhoff catheters. Perit Dial Int. 2011;31:551–557. 20592099 10.3747/pdi.2009.00171

[27] Stegmayr BG Three purse-string sutures allow immediate start of peritoneal dialysis with a low incidence of leakage. Semin Dial. 2003;16:346–348. 12839511 10.1046/j.1525-139x.2003.16061.x

[28] Stegmayr BG Paramedian insertion of Tenckhoff catheters with three purse-string sutures reduces the risk of leakage. Perit Dial Int. 1993;13:S124–S126. 8399546

[29] Dejardin A, Robert A, Goffin E Intraperitoneal pressure in PD patients: relationship to intraperitoneal volume, body size and PD-related complications. Nephrol Dial Transplant. 2007;22:1437–1444. 17308323 10.1093/ndt/gfl745

[30] Ozener C, Bihorac A, Akoglu E Technical survival of CAPD catheters: comparison between percutaneous and conventional surgical placement techniques. Nephrol Dial Transplant. 2001;16:1893–1899. 11522875 10.1093/ndt/16.9.1893

